# The validity of consumer-level, activity monitors in healthy adults worn in free-living conditions: a cross-sectional study

**DOI:** 10.1186/s12966-015-0201-9

**Published:** 2015-03-27

**Authors:** Ty Ferguson, Alex V Rowlands, Tim Olds, Carol Maher

**Affiliations:** Alliance for Research in Exercise, Nutrition and Activity (ARENA), Sansom Institute, University of South Australia, GPO Box 2471, 5001 Adelaide, Australia

**Keywords:** Actigraphy, Physical activity, Sleep, Validity, Triaxial accelerometer, Activity monitor

## Abstract

**Background:**

Technological advances have seen a burgeoning industry for accelerometer-based wearable activity monitors targeted at the consumer market. The purpose of this study was to determine the convergent validity of a selection of consumer-level accelerometer-based activity monitors.

**Methods:**

21 healthy adults wore seven consumer-level activity monitors (Fitbit One, Fitbit Zip, Jawbone UP, Misfit Shine, Nike Fuelband, Striiv Smart Pedometer and Withings Pulse) and two research-grade accelerometers/multi-sensor devices (BodyMedia SenseWear, and ActiGraph GT3X+) for 48-hours. Participants went about their daily life in free-living conditions during data collection. The validity of the consumer-level activity monitors relative to the research devices for step count, moderate to vigorous physical activity (MVPA), sleep and total daily energy expenditure (TDEE) was quantified using Bland-Altman analysis, median absolute difference and Pearson’s correlation.

**Results:**

All consumer-level activity monitors correlated strongly (r > 0.8) with research-grade devices for step count and sleep time, but only moderately-to-strongly for TDEE (r = 0.74-0.81) and MVPA (r = 0.52-0.91). Median absolute differences were generally modest for sleep and steps (<10% of research device mean values for the majority of devices) moderate for TDEE (<30% of research device mean values), and large for MVPA (26-298%). Across the constructs examined, the Fitbit One, Fitbit Zip and Withings Pulse performed most strongly.

**Conclusions:**

In free-living conditions, the consumer-level activity monitors showed strong validity for the measurement of steps and sleep duration, and moderate valid for measurement of TDEE and MVPA. Validity for each construct ranged widely between devices, with the Fitbit One, Fitbit Zip and Withings Pulse being the strongest performers.

## Background

Physical activity has important health benefits, including reducing the risk of cardiovascular disease, some cancers, type-2 diabetes, osteoporosis, anxiety and depression [1-3]. However, many adults are insufficiently active. In Australia, for example, 67% undertake less than 150 minutes of moderate to vigorous physical activity (MVPA) per week [4]. Low-cost techniques that assist people to increase their physical activity are required.

Pedometers have been an effective, low-cost tool, used extensively by researchers, clinicians and individuals to monitor and intervene on physical activity for the past two decades [5]. Conversely, accelerometers have been widely used predominant in research settings to describe physical activity, sedentary behaviour, sleep and total daily energy expenditure (TDEE) [6,7], due to their expense and difficulty of use (requiring proprietary software and expertise for data collection and analysis). In recent years, technological advances have seen the cost of accelerometer-based technology fall, and as a result, the emergence of accelerometer-based devices aimed for the consumer market.

Corporations such as Nike and Fitbit are at the forefront of this market, with wearable technology recognised as a leading technology trend in 2014-15 by many technology commentators and experts [8,9]. Such devices typically cost $USD50-100, making them considerably cheaper than research-grade accelerometers. Many consumer-level devices have displays for immediate feedback and associated free mobile and internet-based applications, providing users with feedback on a variety of metrics including step count, calories burned, stairs climbed, distance travelled, active time and sleep. Some devices also offer the ability to interact with other users via online social networks, which has been shown to have potential benefits for positive health behaviour change [10]. Several manufacturers claim their devices accurately capture activity levels whilst worn on various body sites (e.g. Misfit Shine can be worn on a necklace, wrist band, bra or waist band). Considering these features and flexibility, consumer-level activity monitors, coupled with smartphone technology, have vast potential to enhance user experience and utility [11].

While these new activity monitors offer considerable promise to researchers and clinicians working to assist people to increase their physical activity, monitor energy balance and modify their sleep behaviours, a major limitation to the adoption of these devices in research and clinical settings is the limited scientific evidence regarding their reliability and validity. To date, the Fitbit devices have received the most attention, with a small number of studies scrutinising the validity of various outputs. Dannecker and colleagues [12] examined the ability of the original Fitbit (now twice superseded – first by the “Ultra” and now by the “One”) to measure active energy expenditure among 19 healthy young adults, and found that it underestimated 4-hour energy expenditure by 28% compared with indirect calorimetry (the gold standard physical activity measure). Montgomery-Downs and colleagues [13] found that the original Fitbit overestimated sleep by 67 minutes (SD ± 51) relative to polysomnography. More recently, Takacs and colleagues [14] examined the ability of the Fitbit “One” to count steps during treadmill walking among 30 healthy adults. Participants ambulated at five different speeds for five minutes at each speed, wearing three Fitbit devices (at each hip and in the front pocket of the dominant side). Using direct observation as the criterion, excellent validity (0.97-1.00) and inter-device reliability (99% agreement) were reported, regardless of walking speed or device wear site.

Given the large number of activity monitors now commercially available, methodologies which evaluate them simultaneously are required in order to determine the relative utility of these devices. A recent study by Fulk and colleagues [15] compared the validity of the Fitbit Ultra (now superseded), Nike Fuelband and a traditional pedometer (Yamax SW-701) in people with stroke and traumatic brain injury (n = 50) during a two minute walk test. It was found that the Fitbit Ultra was the most accurate device (95% agreement with direct observation), followed by the Yamax (85%), and the Nike Fuelband (66% accuracy), highlighting that validity can vary widely. Lee, Kim and Welk [16] examined the validity of eight consumer-level devices for estimating energy expenditure in healthy young adults (n = 60). During a 69 minute protocol in a laboratory setting, the consumer-level devices were compared against an indirect calorimetry criterion. The devices were ranked based on percent accuracy, as follows: BodyMedia FIT (90.7% accuracy), Fitbit Zip (89.9%), Fitbit One (89.6%), Jawbone UP (87.8%), Actigraph GT3X (87.4%), DirectLife (87.2%), Nike Fuelband (87%) and Basis BI Band (76.5%). To date, it appears that no studies have scrutinised a large number of devices simultaneously for other variables provided by the devices (e.g. sleep time and MVPA), and no studies thus far have examined the devices in free-living conditions.

This study aimed to address these deficits, by comparing a selection of consumer-level devices against two commonly used research-grade accelerometers in free-living adults. Our hypothesis was that all devices would correlate strongly with the research-grade devices on measures of step count, MVPA, TDEE, and sleep time, and show small absolute differences.

## Methods

### Study design

This study used a cross-sectional design to assess the concurrent validity of consumer-level activity monitors as measures of physical activity and sleep, compared to previously validated research-level accelerometers.

### Consumer-level activity monitors

Seven devices were examined: Fitbit One (Fitbit, Inc., San Francisco, CA, US), Fitbit Zip, Jawbone UP (Jawbone, San Francisco, CA, US), Misfit Shine (Misfit, San Francisco, CA, US), Nike Fuelband (Nike, Inc., Oregon, WA, US), Striiv Smart Pedometer (Striiv, Inc. Redwood City, CA, US), and Withings Pulse (Withings, Issy les Moulineaux, France). Devices were chosen based on those available to the authors for purchase between February and August 2013. The total number of consumer-level devices was capped at seven, based on the feasibility of participants concurrently wearing this number of devices (in addition to two reference devices). All of the activity monitors measure various physical activity parameters, with four also measuring sleep-related parameters (Table 1). Additionally all devices included the option of being worn at the hip and/or the wrist.

**Table 1:** **Device details, set up parameters and analysis software**

**Device**	**Actigraph GT3X+**	**BodyMedia SenseWear**	**Fitbit one**	**Fitbit zip**	**Nike fuelband**	**Jawbone UP**	**Striiv Smart Pedometer**	**Misfit Shine**	**Withings Pulse**
**Released**	Sep 2011	July 2010	Sep 2012	Sep 2012	Feb 2012	Nov 2012	Apr 2012	Aug 2013	June 2013
**Retail Price** (USD)	$249.00	$1417.95	$99.95	$59.95	$149.00	$129.99	$99.95	$119.95	$99.95
**Parameters Measured**									
Steps	✔	✔	✔	✔	✔	✔	✔	✔	✔
Distance	✖	✖	✔	✔	✔	✔	✔	✔	✔
Calories burned	✖	✔	✔	✔	✔	✔	✔	✔	✔
Elevation	✖	✖	✔	✖	✖	✖	✔	✖	✔
Sleep time	✖	✔	✔	✖	✖	✔	✖	✔	✔
Sleep quality	✖	✖	✔	✖	✖	✔	✖	✔	✔
Active time	✔	✔	✔	✔	✔	✔	✔	✔	✔
**Wear site**	Right hip	Left upper arm	Right hip	Right hip	Left wrist	Left wrist	Right hip	Left wrist	Right hip
**Set up parameters**	H, W, Sex, DOB. 80 Hz 1 s epoch LFE off	H, W, Sex, DOB, Handedness	H, W, Sex, DOB	H, W, Sex, DOB	H, W, Sex, DOB	H, W, Sex, DOB	H, W, Sex, DOB	H, W, Sex, DOB	H, W, Sex, DOB
**Set up software**	Actilife v6.6.3	Sensewear Professional 7.0	Fitbit iPhone app v2.0.1	Fitbit iPhone app v2.0.1	Nike + Fuelband iPhone app v2.0.0	UP by Jawbone iPhone app v2.8.1	Inbuilt device software	Shine iPhone app v1.4.0	Withings Health Mate iPhone app v1.21
**Analysis**	Actilife v6.6.3 MVPA (Freedson et al. cut-points) (21) Steps/day	Sensewear Professional 7.0 TEE MVPA = > 3 METs Steps/day	‘Fitbit’ iOS app v2.0.1 and via FitBit online dashboard software	‘Fitbit’ iOS app v2.0.1 and via Fitbit online dashboard software	‘Nike + Fuelband’ iOS app v2.0.0	‘UP by Jawbone’ iOS app v2.8.1	Inbuilt device software	‘Shine’ iOS app v1.4.0	‘Withings Health Mate’ iOS app v1.20 and via Withings online dashboard software

LFE = low frequency extension; H = height; W = weight; DOB = date of birth; MVPA = moderate to vigorous physical activity; MET = metabolic unit.

### Reference devices

The consumer-level devices were compared with two research grade tri-axial accelerometers/multi-sensor devices: BodyMedia SenseWear Model MF (BodyMedia Inc., Pittsburg, PA, USA) and ActiGraph GT3X+ (Actigraph, Pensicola, FL, USA). The reference devices collectively have accepted reliability and validity as free-living measures of physical activity and sleep time. Specifically, the SenseWear has been validated for TDEE under free-living conditions against doubly-labeled water [17-19] yielding strong correlations (ICC = 0.66-0.80) and small biases (–22 to +112 kcal/day). Additionally, SenseWear has been validated as a measure of sleep time compared with polysomnography (epoch-by-epoch agreement = 79.9% [20] and total sleep time r = 0.84 [21]). The GT3X+ has been shown to be a valid measure of both step count compared with observation (percentage error <1.5% [22]; percentage error ≤1.1% [23]; ICC ≥0.84 [24]) and MVPA compared to indirect calorimetry (r = 0.88) [25].

### Study population

A convenience sample of 21 healthy participants was recruited. Participants were eligible for inclusion if they were aged 18 years or over, lived in metropolitan Adelaide, South Australia, and could ambulate without walking aids. Participants were excluded if they experienced an injury or illness affecting their mobility (self-reported).

### Procedure

The University of South Australia Human Research Ethics Committee approved this study and all participants provided informed consent prior to commencing the study. Participants attended an appointment at which demographic data (date of birth, sex and dominant side) were obtained, with height and mass measured following standardized procedures [26]. All devices were set up with unique user accounts using the parameters detailed in Table 1 [27,28].

All nine devices were fitted to the participant in the following locations: SenseWear on the left upper arm; Fuelband, UP and Shine on the left wrist; GT3X+, One, Zip, Pulse and Striiv on the right side of the waist on an elasticised belt. Where consumer-level devices were designed for multiple wear locations, devices suitable for wrist wear were worn on the wrist; otherwise the device was worn on the waist. Placement order of the devices at the wrist and waist was randomised.

Participants were instructed to leave all devices on simultaneously for approximately 48 hours (including sleep, but excluding showering) in order to capture a full overnight sleep episode as well as a to 24-hours of activity data from midnight to midnight. The wear period was not limited to a particular period of the week (i.e. not restricted to weekdays only or weekends only) and no guidelines or restrictions on activity levels or sleep were provided, in order to ensure the study broadly represented free-living conditions. Participants were instructed in how to turn sleep mode on and off for the relevant devices (Shine, Pulse, One, UP). Participants were not given access to any of the device software or account information and were also instructed not to turn off, modify or change any device wear locations once fitted. Devices were collected after the 48-hour wear period. Data collection took place in November-December 2013.

### Statistical analyses

Data relating to physical activity were limited to the full calendar day (24 hour period midnight to midnight) after initialisation. Data relating to sleep were limited to the first night of sleep (24-hour period midday to midday, excluding naps) following initialisation. Data were extracted using the proprietary software for all consumer devices, in the same fashion that a consumer would utilise the software, and were visually checked for outliers. Participants were asked about any non-wear periods, and all indicated full compliance (that is, removal only for bathing). Compliance was checked using the two reference devices (Sensewear, which automatically detects non-wear time, and Actigraph, where we used a criterion of 30 minutes of continuous zero readings), and the data confirmed the participants’ reports.

Participants’ demographic data were analysed descriptively. Device validity was determined for four key constructs: step count (steps/day); MVPA (minutes/day); TDEE (calories/day); and sleep time (minutes/night). Devices were omitted from analysis in a particular construct if the device did not measure that construct.

Step count was determined by comparing the consumer-level activity monitors with the GT3X+. The validity of both TDEE and sleep time were determined by comparing the consumer-level activity monitors with the SenseWear, on the basis that it had best established validity for these constructs out of the two research devices [20,17]. Many of the consumer devices provided outputs for multiple aspects of the physical activity intensity spectrum. After extensive discussion, it was decided that analyses in this study would focus on MVPA, on the basis that it has well established health benefits, is widely reported in the scientific literature, and because it is the focus of public health physical activity guidelines. Validity of MVPA was determined by comparing the consumer-level activity monitors with the GT3X+ [25]. No consumer-level activity monitors explicitly measured MVPA, however Striiv, Shine, UP, One, and Zip all measured either total ‘active minutes’ or breakdowns of active minutes according to intensity (e.g. ‘light’, ‘moderate’ and ‘very’ categories by Fitbit software). A consensus approach amongst the research team was used to determine which consumer-level device outputs most closely reflected MVPA, and the following were agreed on: One: sum of very active and moderate physical activity; Zip: sum of vigorous and moderate physical activity; UP: active time; Striiv: active time; Shine: sum of “kinda”, “pretty” and “very” active.

Validity on all four constructs was quantified using Bland-Altman analysis (bias and limits of agreement), Pearson’s r, and the median absolute difference (MAD) between the research and consumer-level devices. The median absolute difference was used because data were highly skewed.

*A priori* power analyses were undertaken based on existing data on correlations among various research devices, which suggested that the correlation between consumer-level and research devices would be about 0.85. If the actual population correlation between consumer-level and research devices was 0.85, then a target sample size of 21 would yield, in 95% of cases, a sample correlation between 0.65 and 0.94.

## Results

Twenty-one potential participants were approached; all met the eligibility criteria, were available during the study period (November - December 2013), agreed to participate and completed the study. Gender distribution was approximately equal with 10 males (BMI 27.3 ± 3.2) and 11 females (BMI 25.5 ± 5.2), with ages ranging from 20 to 59 years (mean age 32.8 ± 10.2 years). All participants were right hand dominant.

All 21 participants wore the full set of devices for the 48-hour duration, however some data were lost due to data extraction error (7 sets MVPA each for One and Zip), device malfunction (1 set steps each for Fuelband, Zip, Pulse; 1 set TDEE each for Zip and Pulse; 1 set MVPA for Zip; 1 set sleep for Pulse), and participant error (2 sets sleep for One). No data were lost from the two reference devices.

**Figure 1:**
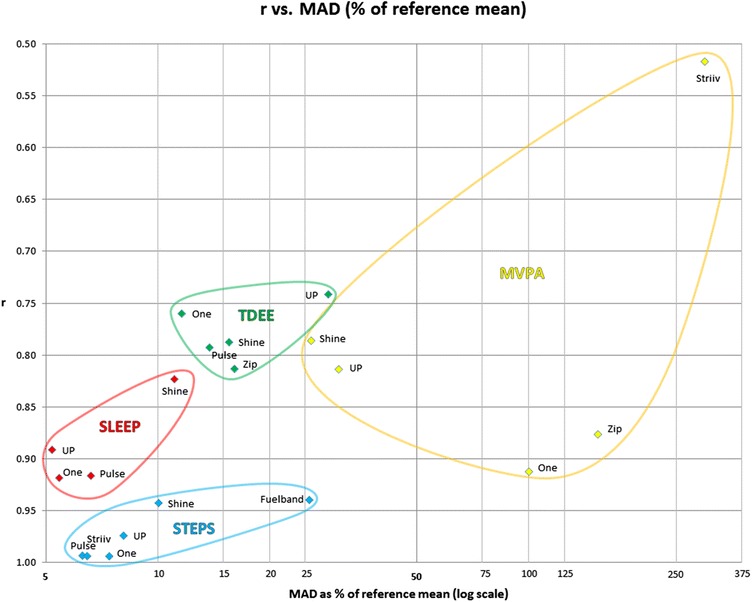
**Scatter-plot of Pearson’s r against the median absolute difference (MAD) as a % of the mean of the relevant reference device.** Note: r = Pearson correlation; MAD = median absolute difference; TDEE = total daily energy expenditure; MVPA = moderate to vigorous physical activity; UP = Jawbone UP; One = Fitbit One; Zip = Fitbit Zip; Shine = Misfit Shine; Pulse = Withings Pulse; Fuelband = Nike Fuelband; Striiv = Striiv Smart Pedometer.

Figure 1 shows a scatterplot of Pearson’s r against the MAD (as a % of the mean of the relevant research device) for all four outputs. Correlations and differences varied between constructs. Steps demonstrated a trend towards having the strongest correlations and smallest differences. This pattern was closely followed for sleep. For TDEE, correlations and differences were modest. Trends were difficult to determine for MVPA, with correlations and differences varying.

Table 2 shows the correlation, MAD, bias, and 95% limits of agreement for steps, MVPA, TDEE, and sleep time assessed by the consumer-level devices relative to the research devices.

**Table 2:** **Means (SD), correlations, median absolute difference, and bland altman output for each device on the constructs of steps, MVPA, TDEE and sleep**

		**Nike fuelband**	**Striiv smart pedometer**	**Misfit shine**	**Jawbone UP**	**Withings pulse**	**Fitbit zip**	**Fitbit one**
**STEPS**	Reference	BS	GT3X+	BS	BS	GT3X+	GT3X+	GT3X+
	Reference mean (SD)	9959 (4844)	10516 (5070)	9959 (4844)	9959 (4844)	10516 (5070)	10516 (5070)	10516 (5070)
	r	0.94	0.99	0.94	0.97	0.99	0.99	0.99
	ICC	0.80	0.95	0.90	0.97	0.99	0.98	0.95
	MAD	2551	679	1002	806	660	447	779
	Range of differences	-5309 to +143	-679 to +1887	-4693 to +1804	-1978 to +2252	-2386 to +832	-970 to +1596	-890 to +1849
	Bias	-2529	675	-1054	-251	-632	464	584
	LoA (U)	910	2089	2288	1889	663	1799	1980
	LoA (L)	-5968	-739	-4395	-2391	-1927	-871	-813
**MVPA (min)**	Reference		GT3X+	GT3X+	GT3X+		GT3X+	GT3X+
	Reference mean (SD)		58.5 (37.6)	58.5 (37.6)	58.5 (37.6)		58.5 (37.6)	58.5 (37.6)
	r		0.52	0.79	0.81		0.88	0.91
	ICC		0.08	0.79	0.70		0.36	0.46
	MAD		174.3	15.2	18.0		89.8	58.6
	Range of differences		+77.0 to +299.3	-79.7 to +36.3	-4.7 to +96.5		+10.0 to +157.2	+1.0 to +137.2
	Bias		190.4	-5.2	22.7		85.7	65.9
	LoA (U)		344.9	45.3	68.2		172.1	154.9
	LoA (L)		35.9	-55.8	-22.7		-0.8	-23.2
**TDEE (kcal)**	Reference			BS	BS	BS	BS	BS
	Reference mean (SD)			3005 (569)	3005 (569)	3005 (569)	3005 (569)	3005 (569)
	r			0.79	0.74	0.79	0.81	0.76
	ICC			0.51	0.27	0.54	0.57	0.55
	MAD			468	866	415	484	349
	Range of differences			-996 to +100	=1937 to -94	-1284 to -58	-1145 to +218	-1724 to -83
	Bias			-479	-898	-533	-497	-475
	LoA (U)			214	-150	170	158	265
	LoA (L)			-1172	-1647	-1236	-1152	-1216
**SLEEP (min)**	Reference			BS	BS	BS		BS
	Reference mean (SD)			423.6 (73.5)	423.6 (73.5)	423.6 (73.5)		423.6 (73.5)
	r			0.82	0.89	0.92		0.92
	ICC			0.71	0.85	0.87		0.90
	MAD			47.0	22.0	28.0		23.0
	Range of differences			-61 to +125	-31 to +132	-17 to +104		-45 to +76
	Bias			44.2	23.5	24.4		15.9
	LoA (U)			135.3	96.2	95.2		82.9
	LoA (L)			-46.9	-49.2	-46.5		-51.0

Note: BS = BodyMedia SenseWear; GT3X + = ActiGraph GT3X+; MVPA = Moderate to Vigorous Physical Activity; MAD = Median Absolute Difference; LoA (U) = Limits of Agreement (Upper); LoA (L) = Limits of Agreement (Lower); TDEE = Total Daily Energy Expenditure.

All of the consumer-level devices measured steps, and correlations with reference devices were very strong (r = 0.94-0.99). Bland-Altman analyses suggested that three of the devices slightly over-counted (Striiv, Zip, One) while four under-counted (Fuelband, Shine, Up, Pulse). Of note, the Fuelband, on average, undercounted daily steps by 2,529 (reference device (SenseWear) mean = 9,959 steps per day).

Five of the consumer-level devices (Striiv, Shine, Up, Zip, One) were considered to measure a parameter similar or equivalent to MVPA time (as defined in section 2.10). Correlations between readings from the consumer-level devices and reference devices ranged from weak to strong (r = 0.52-0.91). Bland-Altman analyses showed large differences between the mean values reported by the consumer-level devices and the reference devices: the Shine, for example, under-counted (mean = 53.3 min of MVPA compared to reference device (GT3X+) mean = 58.5 min), while the Striiv over-counted (mean = 249 min of MVPA compared to reference device (GT3X+)).

Of the five consumer-level devices (Shine, Up, Pulse, Zip, One) that measured TDEE, correlations with the reference devices were moderate to strong (r = 0.74-0.81). Bland-Altman analyses suggest all devices considerably underestimated TDEE compared to the reference device (SenseWear, mean = 3005 kcal), ranging from 475 kcal (One) to 898 kcal (UP).

Of the four consumer-level devices (Shine, Up, Pulse, One) that measured minutes of sleep, all correlated strongly with the reference device (r = 0.82-0.92). Bland-Altman analyses showed all devices overestimated minutes of sleep - most notably, the Shine (mean = 44 min) compared to reference device (SenseWear) mean = 424 min).

## Discussion

This study aimed to examine the validity of a range of consumer-level activity monitors across a range of variables in free-living conditions. In general, the consumer-level devices were highly accurate in measurement of steps, and quite accurate for sleep quantity. However, measures of TDEE and MVPA were less accurate, in general demonstrating moderate to strong correlations with the research-grade accelerometers, but often large MADs. Within each activity construct, the validity of the consumer-level devices varied markedly. Across the domains, the One, Zip and Pulse generally performed strongly.

Steps were generally counted with a high degree of accuracy by the consumer-level activity monitors, though two devices substantially undercounted steps (MADs of 10% for Shine and 26% for Fuelband) and hence underperformed compared to conventional pedometers (such as Yamax; [29]). The findings of the current study, favouring the Fitbit activity monitors, concur with other studies which have similarly found them to be highly valid for measuring step counts in healthy subjects [14], and to perform better than the Fuelband in patients with brain and stroke injury [15]. The findings of the present study extend this previous research by indicating that these devices can accurately measure steps in free-living conditions over longer durations, as opposed to treadmill walking for 25 minutes [14].

One of the advantages offered by some of the consumer-level devices examined in the current study is the ability to quantify sleep. Research-level accelerometers have been shown to be moderate to strong performers for sleep measurement [15,16], however the performance of the consumer-level activity monitors is relatively unknown. In the present study all four of the consumer-level devices that claimed to measure sleep duration performed reasonably well in relation to the research-level accelerometer, but consistently over-estimated sleep duration, and to a reasonably large magnitude (SenseWear; r = 0.82-0.92, MAD = 22-47 min). While little is presently known about the range of consumer-level accelerometers as measures of sleep, this tendency to over-report is similar to what was found in the systematic review. The only consumer-level device identified was the original Fitbit, in Montgomery-Downs et al’s [13] study of 24 healthy adults, where it was shown that, on average, this device significantly over-reported total sleep time compared to a research accelerometer (Actiwatch; by 24 minutes) and polysomnography (by 67 minutes). The extent of over-estimation of sleep duration by the consumer devices is likely to reduce their utility for users, particularly if they are attempting to compare their sleep duration to external benchmarks (e.g. 6 - 8 hour sleep guidelines).

Five devices measured TDEE. In general, validity for devices related to TDEE was moderate to strong (r = 0.74-0.81; MAD = 12-29%). Of note, the two Fitbit devices (One and Zip) were superior to the Up when compared to the reference device. This is consistent with the findings of Lee, Kim and Welk [16] when comparing a range of consumer-level devices to the gold standard measure of indirect calorimetry. Similar to Lee, Kim and Welk’s [16] findings, all devices in the present study underestimated TDEE. Dannecker et al. [12] also explored the validity of a superseded Fitbit device and found that it underestimated energy expenditure (compared to indirect calorimetry) by almost twice as much as the Fitbits in Lee, Kim and Welk’s [16] study and the present study, suggesting that this variation might be a reflection of device accuracy improving with model updates.

Finally, all of the devices measured some aspect of physical activity duration, although the way this was classified tended to vary and specific information regarding intensity cut-points was not provided by the manufacturers. The correlation-based analyses showed weak to strong correlations with the reference devices (r = 0.52-0.91), however MADs were large (e.g. in the best case, the Shine underestimated MVPA by 15 minutes a day, or 26%, while the Striiv overestimated physical activity by 190 minutes a day, or 325%, both relative to the GT3X+). While MVPA is generally accepted as a yardstick of healthful physical activity in the research field, many of the devices provided feedback on multiple physical activity variables, and none were explicitly identified as MVPA, so the discrepancies here may arise both from definitional and measurement issues. Even within the accelerometry research field, there is considerable debate about how MVPA should be operationalised, with various cut-points proposed by different experts, leading to vastly different daily values and difficulty in comparing across studies [30]. Given this, it is suggested that the best interpretation of findings is that the weak to strong correlation coefficients are a preliminary indicator of reasonable validity of some of the consumer-level devices, and that the poor MAD values are probably reflective of issues with operationalisation. With no previous studies exploring the validity of these devices for measuring MVPA, further research is warranted.

Strengths of the current study are that the devices were tested in free-living conditions (the environment they are designed for) over a whole (24 hour) day. Additionally, a wide range of consumer and reference devices were used, allowing for comparison of the validity between devices. This represents a significant contribution over previous research into consumer accelerometers, which has tended to be laboratory-based and, in many cases, has only examined a small number of devices. Furthermore, this study examined several different variables collected by the devices (namely, steps, sleep quantity, TDEE and physical activity), giving a more complete picture of their capabilities.

The study has a number of limitations, which should be acknowledged. The number of consumer devices scrutinised in this study was limited to seven due to concerns around participant burden. It was beyond the scope of the study to examine all consumer devices available on the market. In addition, further devices have entered the market subsequent to data collection. Incomplete data sets were obtained for some variables (in particular, MVPA for the One and Zip) due to data extraction error, device malfunction and participant error, and caution should be taken when interpreting findings for these particular results. The study protocol did not examine reliability and, due to being undertaken in free-living conditions, convergent validity, rather than criterion validity, was scrutinised. Some of the consumer-level devices purported to measure variables that none of the research devices measured (e.g. stairs ascended and sleep quality), thus they could not be assessed in the current study. While some of the consumer-level devices are promoted as being able to be worn on a variety of body sites, this study only examined the validity of such devices at one site, and it is important to note that validity is likely to vary at the different body locations.

The fact that moderate to strong correlations were produced for virtually all devices and variables provides preliminary evidence that all of the devices are reasonably valid for measuring the respective variables. However, the real world implications of the mediocre MAD results are mixed. It could be argued that so long as the device is reliable, it will provide users with sufficient feedback to successfully gauge and modify their behaviour. However, if consumers are using the devices to compare their behaviour to external benchmarks (e.g. physical activity or sleep guidelines) the inaccuracy experienced with some of the devices is likely to be a source of frustration. In particular, inaccuracies with the TDEE estimates suggest that these devices will be of little use to someone attempting to use the data to balance energy expenditure with energy intake.

For researchers considering using these devices, based on current evidence, it would appear that the Pulse and the two Fitbit devices (One and Zip) were the stronger performers. Validity did not reflect price, with the most expensive device (Fuelband) being one of the weakest performers, and the cheapest device (Zip) one of the best.

As a tool for objectively measuring physical activity in observational studies, the consumer-level devices risk participant reactivity (as they provide feedback to the wearer), a recognised issue with traditional pedometers. These consumer-level devices perhaps offer greatest potential as intervention tools. Particularly, the fact that they provide feedback on a range of variables makes them appealing for lifestyle interventions (e.g. simultaneously targeting physical activity and sleep). Additionally, the software associated with many of these devices offer new opportunities, such as the ability for researchers to monitor activity in real-time via the internet. Additionally, these devices tend to be stylish, unobtrusive and versatile, which may enhance participant wear time compliance.

Future research examining other aspects of the consumer-level devices, such as their reliability, acceptability, usability and durability are warranted. A challenge for the research in this field will be keeping pace with the rapidly evolving consumer market. Devices are being developed at an extraordinary rate, generally without any published data on validity. The general public are using these devices to make decisions about physical activity and sleep. They may also offer good alternatives for more expensive and cumbersome research-level devices. All too often, by the time devices can be scientifically evaluated and results published in peer-reviewed process, the consumer market may have moved on, with new models, new devices and software updates being released continually.

## Conclusion

The new wave of consumer-level activity monitors offers exciting possibilities for individuals, clinicians and researchers. Our study offers preliminary evidence for their validity in measuring steps, and perhaps also sleep duration, with the Fitbit One, Fitbit Zip and Withings Pulse strong performers. Future research scrutinising the devices’ reliability and usability are warranted, though keeping pace with the rapidly evolving consumer-level activity monitor market will be a challenge.
